# A predictive model towards understanding the effect of reinforcement agglomeration on the stiffness of nanocomposites

**DOI:** 10.1177/00219983221076639

**Published:** 2022-03-21

**Authors:** Eyup Can Demir, Abdelhaq Benkaddour, Daniel R Aldrich, Mark T McDermott, Chun Il Kim, Cagri Ayranci

**Affiliations:** ^1^Department of Mechanical Engineering, 120449 University of Alberta Faculty of Engineering, Edmonton, AB, Canada; ^2^Department of Chemistry, 3158 University of Alberta, Edmonton, AB, Canada

**Keywords:** Polymer nanocomposites, elastic behavior, modeling, probabilistic methods, transmission electron microscopy

## Abstract

Nanocomposite technologies can be significantly enhanced through a careful exploration of the effects of agglomerates on mechanical properties. Existing models are either overly simplified (e.g., neglect agglomeration effects) or often require a significant amount of computational resources. In this study, a novel continuum-based model with a statistical approach was developed. The model is based on a modified three-phase Mori–Tanaka model, which accounts for the filler, agglomerate, and matrix regions. Fillers are randomly dispersed in a defined space to predict agglomeration tendency. The proposed model demonstrates good agreement with the experimentally measured elastic moduli of spin-coated cellulose nanocrystal reinforced polyamide-6 films. The techniques and methodologies presented in the study are sufficiently general in that they can be extended to the analyses of various types of polymeric nanocomposite systems.

## Introduction

Nanocomposites have been extensively investigated for use in various applications, such as wearable sensors, flexible electronics, soft robotics, blood vessel prosthesis, and bone tissue scaffolds.^1–6^ The high surface-to volume ratio of nanofillers enables significant enhancement of mechanical properties even at low loading which opens pathways to high-performance, lightweight materials. The addition of nanofillers can alter the polymer crystallinity, crystalline morphology, or chain conformation and thus influence the mechanical response.^7^ While understanding a number of variables is of critical importance, conventional composite theories assume that each phase maintains its initial properties, and the addition of nanofillers does not change the properties of the matrix. Researchers have modified conventional theories by introducing dominant parameters (i.e*.*, interface, agglomeration, and shape of nanofillers) to predict mechanical response.^8–12^ However, the cohesive forces between nanoparticles become dominant and lead to non-uniform filler dispersion, that is, particle agglomerations, especially at higher concentrations of nanofillers. Agglomerates result in inefficient load transfers and localized stress concentration sites that dramatically reduce the mechanical properties of nanocomposites. As a result, agglomeration of the nanofiller inhibits the expected promise of nanocomposites.^13–17^

It is important to explore and model the effects of agglomerates on the mechanical response of nanocomposites to achieve the benefit of nanofillers’ properties. Conventional continuum-based models (*e.g*., Reuss, Voigt, and Halpin–Tsai Models) are often utilized and/or modified to predict the stiffness of short-fiber composites ^7,13,14^ via homogenization of two phases.^15–18^ These models presume uniform distribution of fillers, uniform stress or strain throughout the composite, and perfect bonding.^17,18^ However, these assumptions potentially oversimplify the state of composite systems and thus often fail to predict the general response of nanocomposites. For instance, the conventional models predict a linear or exponential increase in elastic modulus of composites with respect to the concentration of fillers; however, experimental observations indicate that the elastic modulus of nanocomposites reaches a plateau value or decreases with increasing loading.^19–23^ It is often observed that conventional models predict the elastic modulus at very low concentrations and often diverge from experimental findings as nanofiller concentration increases.^19,24–26^ Consideration of the individual state of agglomerates is necessary for accurate predictions at high concentrations, which can be achieved by combining the continuum-based models and statistical approaches.

In the present study, we address deficiencies in current models with a three-phase analytical Mori–Tanaka model that incorporates agglomerate, free filler, and matrix phases for the prediction of the elastic modulus of nanocomposites. In particular, a statistical approach, based on a Monte Carlo method is used to achieve natural distributions of fillers within the composite. A hierarchical clustering method based on machine learning is integrated into the model to automate the detection of agglomerates and to decrease the execution time of the code. The model is capable of investigating the effect of key variables such as aspect ratio, orientation and distribution of filler, the volume fraction of agglomerate, and material property of each constitute. The model is applied to cellulose nanocrystal (CNC) reinforced polyamide-6 (PA6) nanocomposites, and it can be used to understand and study the effect of agglomerates in nanocomposites and predict their mechanical properties.

## Modeling

A polymer nanocomposite, consisting of two constituents (*i.e.,* a filler and matrix), can be described by multiple phases/regions including filler, interface, agglomerate, and matrix. The interface and agglomeration phases have been studied in literature.^9,27,28^ We expand the previous studies on agglomeration by developing a model that considers three phases: matrix phase (matrix-only region), free/individual filler phase (filler-only region where no agglomerate exists), and agglomerate phase (more than one filler particle surrounded by matrix). Each phase is analyzed separately and then homogenized to calculate the stiffness of a nanocomposite. The matrix and free filler phases can be accommodated by using the Mori–Tanaka model (see ^29^ and derivation therein). Thus, two critical points remain to be investigated and developed: *distribution of fillers* and *definition of agglomerates*. We used the Monte Carlo method and defined the parameter *“critical distance”* to cover the former and latter, respectively.

### Filler distribution

**Figure 1.**
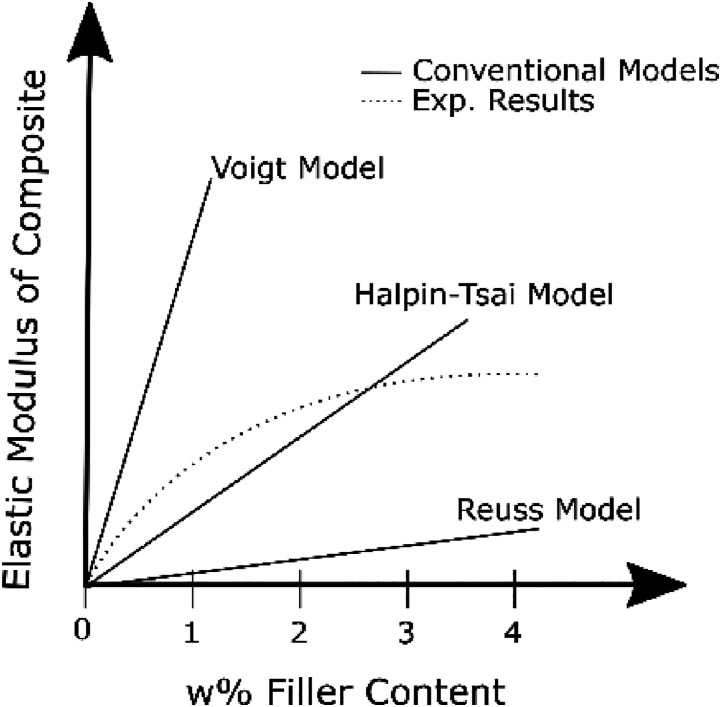
Schematic illustration of different conventional models and experimental results.

It is almost impossible to obtain a perfect dispersion of fillers; therefore, agglomerates are prevalent in many nanocomposites. This inhomogeneity is one of the main reasons why the aforementioned conventional models struggle to predict experimental results of many nanocomposite systems. Commonly observed elastic modulus vs. % filler trends for nanocomposites are shown in Figure 1.^30–32^

Conventional models generally agree with experimental results at low loadings (∼0.5–1 w%); however, as filler content increases, the model predictions often fail. The introduction of a simple methodology into the model to computationally account for randomly distributed reinforcements in a *defined space* could be a powerful tool.

**Figure 2.**
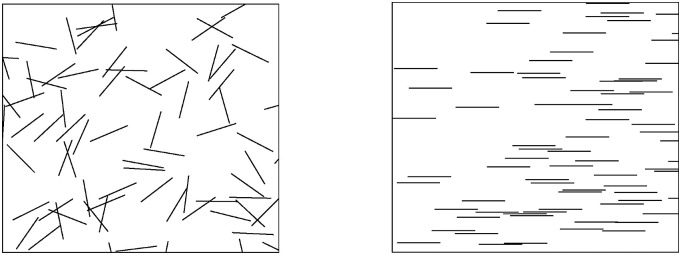
Schematics of the defined space representing RVE for randomly oriented (a) and aligned fillers (b).

The dispersion of fillers is performed in a two-dimensional space because it is computationally efficient and can represent a slice of three-dimensional space. Numerous studies address dispersion and agglomeration based on two-dimensional scanning electron microscopy (SEM) and transmission electron microscopy (TEM) images.^33–39^ The defined two-dimensional space can represent filler dispersion in the nanocomposite’s repeating volume element (RVE). The number of fillers in the RVE is calculated based on their volume fraction, and then, fillers are distributed in the defined space. The model presented here determines the fillers' locations using the Mersenne Twister algorithm, a pseudorandom number generator, in MATLAB code, and the center of fillers represented by filled circles in the RVE. For the pseudorandom number generation, a uniform distribution was followed for the current study. Various distributions can be observed experimentally and may need to be adopted for different cases. The code is written to avoid overlapping circles. The schematics of anticipated RVEs for randomly oriented and aligned fillers are given in Figure 2(a) and (b), respectively. The effect of filler orientation is captured in the Mori–Tanaka model, and the center of fillers is used to define agglomeration in the simulation.

### Definition of an agglomerate

**Figure 3.**
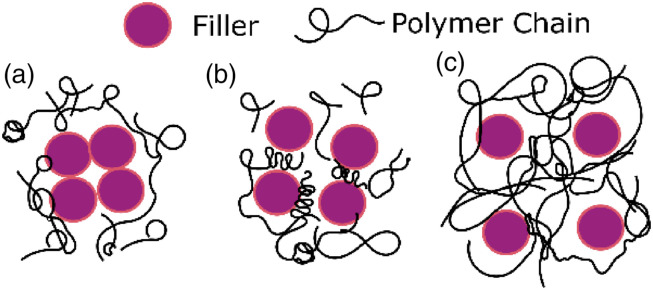
Three states of fillers: contact agglomerated fillers (a), bridge agglomerated fillers (b), and free/homogeneous distributed fillers (c), adopted from Ref. 37.

In literature, distances between fillers are measured and converted into a distribution map to quantify the state of agglomeration.^33,38,39^ However, a clear definition of an agglomerate remains absent in the composite community. Schweizer et al. defined three states: contact aggregation, bridging, and steric stabilization.^40^ Later, Liu et al.^37^ developed the three organization forms of fillers shown in Figure 3 to establish the dispersion state of fillers.^37^

Recent developments in small-angle X-ray scattering technology make it possible to predict fillers’ size, shape, and distribution in soft polymeric materials. Musino et al.^41^ correlate rheological measurements to SAXS analysis to determine at which concentration percolation threshold is achieved. Based on the calculated percolation threshold, they define *aggregates* in the system. These studies guided us in the current work to define the boundaries of agglomerates and establish how to identify agglomerated filler particles. For example, despite the difference in particle proximity between contacted agglomerates and bridge agglomerates shown in Figure 3, the latter should be considered as agglomerates because the strain fields of two closely spaced fillers can intersect. As a result of this intersection, load transfer from particle to matrix would be different from individual homogeneously distributed fillers. To our knowledge, from a mechanical point of view, there has not been a study to validate the distance between fillers in vicinity to define them as an agglomerate.

**Figure 4.**
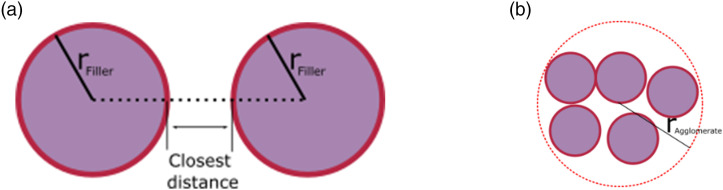
The closest distance between two fillers (a) and agglomerated fillers (b).

Clustering and classification problems have been addressed by data science and various clustering algorithms. The agglomerative hierarchical clustering method, a machine learning method, can be adopted to determine agglomeration of nanofillers. In this method, each object represents a cluster and then, clusters are merged until the desired cluster structure is obtained.^42,43^ In this study, fillers are considered as objects and the distance between them is a parameter to merge clusters. The *critical distance* parameter *γ[D]* is introduced to determine if two or more fillers are agglomerated and it is defined as multiplication of filler diameter. For example, the notation *γ[D]* = 1.5 corresponds to 1.5 times the diameter of the filler (*D*). When the distance between the surfaces of two fillers is less than the *critical distance*, they are considered an agglomerate, given in Figure 4(a). Based on the location of the fillers, the proposed code categorizes the distributed fillers as agglomerated or free fillers. Each filler is labeled with numbers; however, agglomerated fillers were labeled with the same number so that the user can understand if agglomerates exist in the simulation. In doing so, we are able to analyze free fillers, agglomerates, the number of agglomerates, and the size of agglomerates.

We use *“single linkage”* and *“cluster”* functions in MATLAB. The “*single linkage*” function calculates the “*Euclidean distance*” between fillers and uses it as an input for grouping purpose. The “*cut-off*” parameter, under the “*cluster*” function, is then used to label fillers as agglomerated if the closest distance between them is shorter than the *critical distance*. When agglomerates are detected, an imaginary circle is created as a boundary around agglomerated fillers to calculate the volume of each agglomerate (Figure 4(b)). The number of filler particles within each agglomerate is counted and the volume fraction of fillers is determined. In summary, we are able to detect and record the locations and filler fractions of each agglomerate, the number of fillers within each agglomerate, and the ratio of agglomerated filler to free filler.

The experimental observations and analysis in the literature^10,33,38,39,44–46^ show that the properties of agglomeration are unknown, and many researchers correlate agglomerate presence with lower stiffness values in composites. The Reuss model, equation (1), was proposed as a reasonable model to calculate the lower range of stiffness of agglomerated regions

(1) $$\frac{1}{E_{a}}=\frac{v_{m}}{E_{m}}+\frac{v_{f}}{E_{f}}$$

where $E_{a}$ is the elastic modulus of the agglomerate, $E_{m}$ is the elastic modulus of the matrix, and $E_{f}$ the elastic modulus of the filler, $v_{m}$ the volume fraction of the matrix (for agglomerates, $v_{m}$ is the matrix within the agglomerate), and $v_{f}$ the volume fraction of the filler (for agglomerates, $v_{f}$ is the filler within the agglomerate).

### Three-phase Mori–Tanaka model

Eshelby’s inclusion problem investigates stress, strain, and displacement fields both in the inclusion and the matrix.^47^ Mori and Tanaka^29^ applied Eshelby’s solution for an ellipsoidal inclusion problem in order to relate local average stress in the matrix to the transformation strain in the inclusions.^29^ The established Mori–Tanaka model has been improved by many researchers. Tandon and Weng^48^ investigated the effect of the aspect ratio of the reinforcing inclusion with various geometry and established a closed form of the Mori–Tanaka model.^48^ In this work, a three-phase Mori Tanaka model was used based on Benveniste’s work.^49^ The main equations to obtain Benveniste’s closed form of the Mori–Tanaka model are given below. ***T,*** $\bar{t}$ , and *t* represents second-order tensor, vector, and scalar value, respectively. Subscript letters are the constitutes of the system. Strain in the inclusion is related to strain in the matrix with equation (2)

(2) $$\bar{\varepsilon_{f}}=A_{f}\bar{\varepsilon_{m}}$$

where $\bar{\varepsilon_{f}}$ and $\bar{\varepsilon_{m}}$ are the uniform strain vectors in the filler and matrix, respectively, and $A_{f}$ is the strain concentration tensor given by equation (3)

(3) $$A_{f}=\left[I+S_{f}{\left(C_{m}\right)}^{-1}{\left(C_{f}-C_{m}\right)}^{-1}\right]$$

where I is the identity tensor, $S_{f}$ is the fourth order Eshelby tensor, which depends on geometry and poison’s ratio, $C_{m}$ is stiffness tensor of the matrix, and $\;C_{f}$ is stiffness tensor of the filler. Stiffness tensor of the composite can be obtained by equation (4)

(4) $$C=\left(v_{m}C_{m}+v_{f}C_{f}A_{f}\right){\left(v_{m}I+v_{f}A_{f}\right)}^{-1}$$

The Mori–Tanaka model was developed for the unidirectional aligned composites; however, one can introduce orientation averaging tensor for randomly aligned fillers, described in Ref. 45 to calculate the stiffness of composites, which is given by equation (5)

(5) $$C=\left(v_{m}C_{m}+v_{f}\left\{C_{f}A_{f}\right\}\right){\left(v_{m}I+v_{f}A_{f}\right)}^{-1}$$

where the curly brackets {} stands for the indication of orientation averaging, given by equation (6). Subscript letters in equation (6) represent indices rather than the constitutes

(6) $$\left\{M_{ijkl}\right\}=\frac{1}{2\pi }\overset{2\pi }{\underset{0}{\int }}\overset{2\pi }{\underset{0}{\int }}M_{ijkl}\left(\theta ,\varphi \right)\sin\;\varphi \;d\varphi d\theta$$

In a similar way, three-phase Mori–Tanaka (free filler, agglomerate, and matrix phases) can be established by equation (7)

(7) $$C=\left(v_{m}C_{m}+v_{f}\left\{C_{f}A_{f}\right\}+v_{a}\left\{C_{a}A_{a}\right\}\right){\left(v_{m}I+v_{f}A_{f}+v_{a}A_{a}\right)}^{-1}$$

where $C_{a}$ is stiffness tensor of the agglomerate, $A_{a}$ is the strain concentration tensor for the agglomerate, and $v_{a}$ is the volume fraction of the agglomerate. The strain concentration tensor for the $A_{a}$ is calculated by equation (8)

(8) $$A_{a}=\left[I+S_{a}{\left(C_{m}\right)}^{-1}{\left(C_{a}-C_{m}\right)}^{-1}\right]$$

where $S_{a}$ is the fourth order Eshelby tensor for an agglomerate. In this study, agglomeration formation is assumed to be spherical because of minimum surface energy configuration, mechanical stability, and previous studies in the literature.^46,50–52^

### Monte Carlo method

The Monte Carlo method is utilized to estimate the outcome of an uncertain event by generating a large number of likely outcomes. The method calculates possible results by leveraging a probability distribution and repeating the calculations/runs with various inputs. In our study, the uncertain event is the filler dispersion and the outcome is the composite modulus. For accurate composite modulus predictions, we expect to obtain statistically reliable outcomes where reliability measures the reproducibility results with repeated trials.^53^ We set the number of trials in our study to one hundred considering the near infinite number of possible combinations and reasonable processing time.

**Figure 5.**
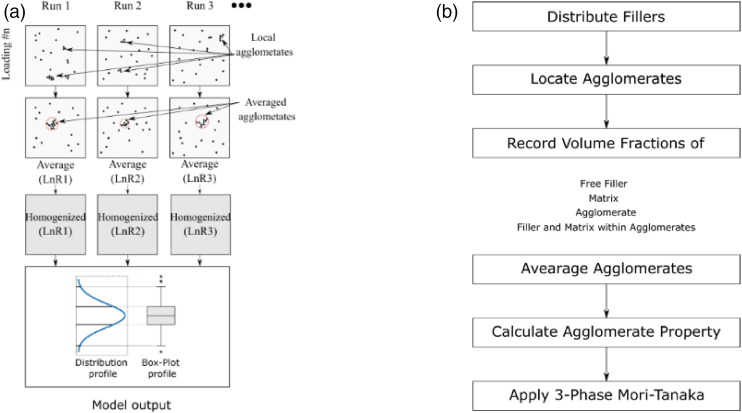
Schematics (a) and steps (b) of the workflow.

As soon as the fillers were dispersed in the defined space, the MATLAB code saved the locations of fillers and the volume fraction of fillers for each agglomerate. The code extracted this information and averaged agglomerates based on their volume as if there was a single agglomerate in the composite. In the end, the nanocomposite contains three phases: the free fillers, the averaged agglomerate, and the matrix. The volume fractions and properties of constitutes obtained are utilized in the three-phase Mori–Tanaka model to calculate the stiffness of the nanocomposite. Figure 5(a) exhibits schematics of the workflow to calculate the stiffness of a nanocomposite for a certain number of fillers. Here, Ln represents the loading number (concentration of the filler), and R1 represents the first run of the simulation. After homogenization, box-plots are used to present the output of one hundred runs. Figure 5(b) summarizes the steps/algorithm of the workflow for one run.

## Experimental method

### Materials and manufacturing

Polymer nanocomposite samples were manufactured with a semicrystalline thermoplastic polyamide-6 (PA6, poly(hexano-6-lactam)-*(C*_*6*_*H*_*11*_*NO)*_*n*_) matrix, and sulfonated cellulose nanocrystals (CNC, (*C*_*6*_*H*_*10*_*O*_*5*_*)*_*n*_) filler. PA6 pellets (Sigma Aldrich) had a density of 1.084 g/mL at 25°C and transition temperature (T_g_) of 62.5°C. CNC was received in spray-dried powder form from CelluForce. The density of CNC is taken as 1.5 g/cm^3^.^54^ Materials were used as received without any further treatment. The samples were prepared on glass substrates via the spin coating method. First, CNC and PA6 were dried at 80°C for 24 h before any suspension preparation. Then, the dried PA6 was dissolved in formic acid, 98% Sigma Aldrich, (the ratio of PA6/formic acid was 20 w/v%) by using a batch sonicator. After complete dissolution of PA6, CNC was added to the suspension based on the designed concentration, and the suspension was kept under agitation until the CNC was dispersed. The prepared suspensions were sonicated one more time before the spinning process for 45 min to disperse CNC within the matrix.

**Figure 6.**
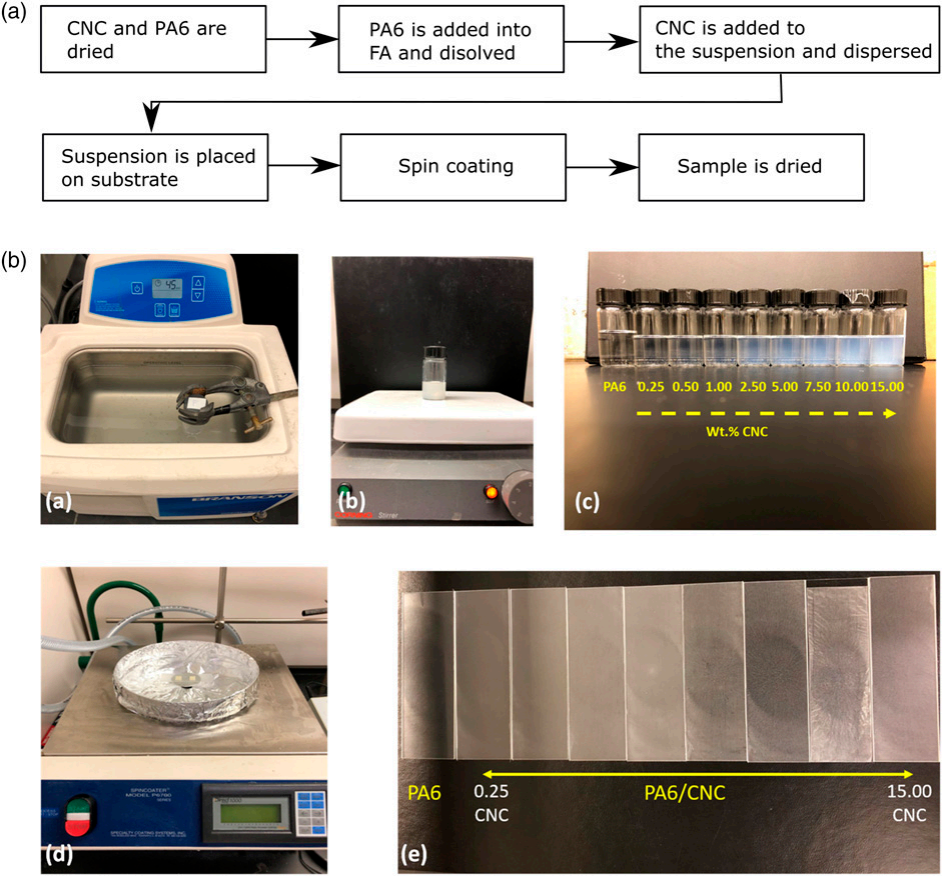
Flowchart (A) and photos (B) of PA6 dissolution in formic acid (a), dissolution of CNC in PA6/formic acid suspension (b), prepared suspensions before the second sonication of CNC/PA6/formic acid (c), spin coating (d), and obtained thin films on glass substrate (e).

Two milliliters of the suspension were placed on a rectangular (75 mm x 25 mm) glass substrate. Then, it was accelerated to 2000 rpm in 15 s and spun at 3000 rpm for 30 s. After the spinning process, the film was left for around 5 min for any remaining solvent to evaporate. The manufacturing steps with a flowchart and photos are given in Figure 6. Samples contain different CNC concentrations in PA6 that vary from 0.0 to 15.0 w%.

### Characterization

#### Transmission electron microscopy (TEM)

Two TEMs (Philips 410 transmission electron microscope and JEOL JEM-ARM200CF S/TEM) were utilized at 100 kV accelerating voltage to understand the morphology, aspect ratio of the CNC, and agglomeration. Two different protocols were followed to prepare the samples of CNC and nanocomposites. In the first protocol, the CNC was dispersed in water (2.5 mg/100 mL), and 0.5 mL of solution was dropped on a TEM copper grip. The CNC was stained with phosphotungstic acid to increase the contrast. The stain solution was dropped on the grid, and excess of it was removed after 10 s. The second protocol involved embedding the nanocomposite sample into epoxy. A thin section (∼120 nm) was microtomed along the longitudinal side of a piece of the sample, using a glass knife in a Reichert-Jung Ultracut E ultramicrotome. The microtomed samples were double-side stained with the extra-long protocol; uranyl acetate—2.5 h and lead citrate for 1 h before the TEM analysis. All images were analyzed using the open source ImageJ software.

#### Tensile test

ASTM 882-12 standard was followed for testing the films and reporting mechanical properties. Produced films, ∼5 μm thick, were cut into 10 mm × 75 mm rectangles by using a rotary cutter and a 3D printed custom cutting plate. The samples were stored overnight in a desiccator before testing. A C-shaped paper support was used to insert samples into the grips, prevent them from sliding from the grips, and reduce stress concentrations at the grips. TA Instrument ElectroForce 3200 with 10 N load cell was utilized. The distance between the grips was 50 mm. The grips were tightened to 4-in.lb torque. The paper support was cut from the middle before the test, and the samples were tested with 0.083 mm/sec rate. A MATLAB code was developed to calculate the elastic modulus and measure the strength and strain-at-break of the composite. While non-uniform strain distribution is a concern due to the grip effect, using an extensometer is a challenging process for ∼5 μm thick films. As allowed in the ASTM 882-12 standard, the aforementioned 50 mm grip distance was the gage length in this study.^55^

## Results and discussion

### TEM analysis of CNC particles and nanocomposites

**Figure 7.**
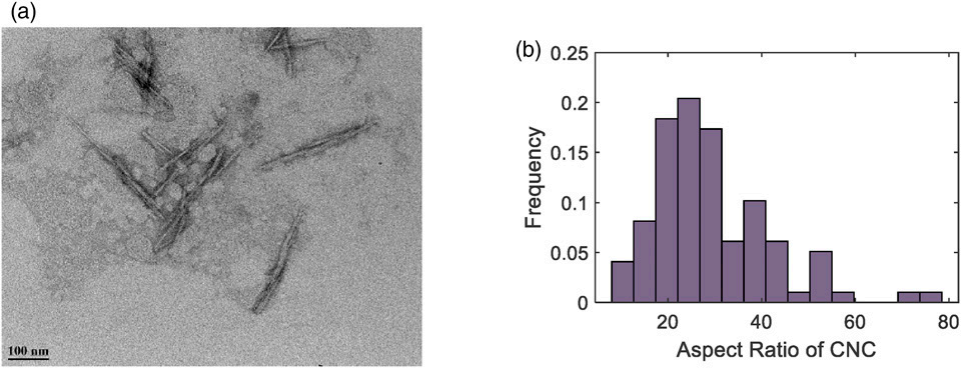
TEM image of CNC (a) and measured aspect ratio (b).

The filler used in this work is CNC. Figure 7(a) exhibits TEM image of fiber like CNC fillers. The aspect ratio (length/diameter) determined by image analysis was found to vary between 11 and 78, and its distribution is given in Figure 7(b). The average length of the particles was determined to be 152.0 ± 49.9 nm and average diameter is 6.0±3.4 nm. The average aspect ratio was calculated as 29.2 ± 12.6 for 100 particles from four different images. The filler and matrix were assumed to be isotropic, and their Poisson’s ratio was set to 0.35 for the model.

**Figure 8.**
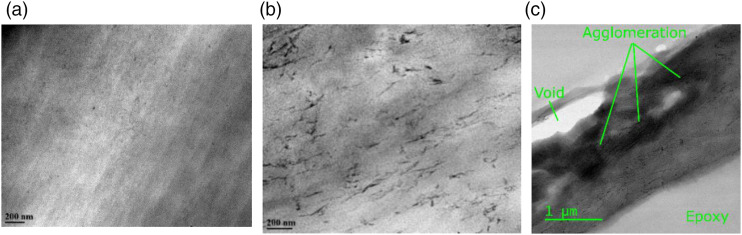
Side view TEM images of a neat PA6 film (a), 15.0 w% CNC reinforced PA6 (b), 15.0 w% CNC reinforced PA6 at lower magnification (c).

TEM was also used to observe the CNC distribution and orientation in the composite. Figure 8(a) and (b) show images of neat PA6 and PA6 containing 15.0 w% of CNC, respectively. We assign the darker fiber-like structures in Figure 8(b) to the CNC due to their size, morphology, and aspect ratio. In addition, the darker structures are not observed in Figure 8(a), supporting their assignment are the CNC particles. A closer inspection of Figure 8(b) reveals a small number of agglomerates with a size generally under a micron. For example, fillers in the top left corner of Figure 8(b) can be assumed to be agglomerated because they are already touching.

The lower magnification of the PA6-CNC composite in Figure 8(c) reveals micron-size agglomerates. These large agglomerates result in a rougher surface and some voids at the interface of the epoxy and sample. Polymer matrix can be observed squeezed between the CNC fillers in the micron-size agglomerates in Figure 8(c). These matrix regions validate our approach that agglomerates consist of matrix and filler. Further, from Figure 8(c), we can conclude that agglomerates converge to a spherical shape compared to the rod-shaped free fillers.

**Figure 9.**
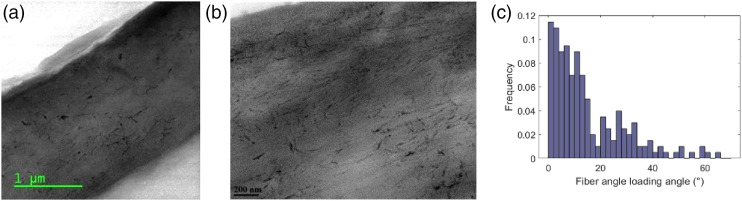
TEM images for filler orientation low (a) and high (b) magnification. Distribution of the angle w.r.t. the testing direction (c).

The orientation of fillers is critical for the model and mechanical response of the sample. Our samples were prepared by spin-coating, which can influence the orientation if the CNC particles. Figure 9(a) and (b) show low and high magnification TEM images that were analyzed to obtain an orientation distribution of the fillers. The angle of orientation of two hundred particles was measured with respect to the longitudinal direction of the specimen. Figure 9(c) shows the distribution of the angle of deviation from the testing direction. Hence, we conclude that around 70% of the fillers deviate from the longitudinal direction by only up to 20°; therefore, the free fillers were assumed aligned for our modeling purposes.

### Uniaxial tensile tests

**Figure 10.**
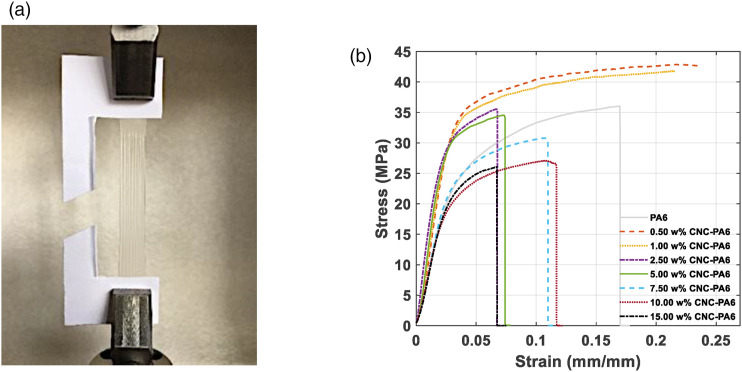
Photo of a sample during a tensile test (a) and representative stress-strain curves for each sample type (b).

Nanocomposite samples of PA6 containing eight different CNC concentrations (0, 0.5, 1.0, 2.5, 5.0, 7.5, 10.0, and 15.0 w%) were prepared and at least five samples from each type were tested. Figure 10(a) and (b) show a photo of a sample under the tensile test and a representative stress-strain curves for each type of the samples. The white paper, seen in Figure 10(a) was used to lower stress generated at the grips and prevent sliding of the films from the grips. In general, the addition of CNC less than 5 w% enhanced the stiffness, yield and ultimate strength, and lowered the strain-at-break compared to neat PA6.

Table 1 lists the elastic modulus, tensile strength, and strain-at-break values of the neat PA6 and PA6 nanocomposite samples. According to the one-way ANOVA test, there is a significant difference in the average elastic modulus, tensile strength, and strain-at-break between the 8 groups (0–15 w% CNC PA6) (F(7,35) = 19.61, p < 0.001). Two-tail T-tests were performed to order the groups and to show a relative significant differences between groups.

**Table 1.** Mechanical properties of nanocomposites at different CNC w% in PA6.

CNC w% in PA6	0	0.5	1.0	2.5	5.0	7.5	10.0	15.0
Elastic modulus (MPa)	911.0 ± 233.9^c^	1366.0 ± 208.6^ab^	1307.9 ± 257.5^ab^	1627.9 ± 107.3^a^	1556.3 ± 124.6^ab^	913.3 ± 128.4^c^	949.9 ± 140.1^c^	821.0 ± 124.5^c^
Tensile strength (MPa)	38.8 ± 1.4^b^	44.9 ± 1.9^a^	43.6 ± 1.4^a^	38.7 ± 2.0^b^	38.2 ± 1.6^b^	32.4 ± 1.2^c^	28.2 ± 0.6^d^	25.9 ± 0.9^e^
Strain-at-break	0.207 ± 0.021^ab^	0.195 ± 0.043^b^	0.214 ± 0.010^a^	0.092 ± 0.036^c^	0.100 ± 0.018^cd^	0.139 ± 0.036^c^	0.123 ± 0.027^c^	0.081 ± 0.018^d^

The superscripts (a, b, c, d, and e) indicate the statistically important differences in the data sets in each row (*p* < 0.05), as determined through one-way ANOVA analysis and T-test. The superscripts are ordered alphabetically, and the largest mean comes the earliest in the alphabet.

The average elastic modulus of neat PA6 was measured as 911.0 MPa (StDev = ± 233.9 MPa). The addition 0.5 w% CNC increased the elastic modulus of the composite to 1366.0 MPa (StDev = ± 208.6 MPa), which corresponds to approximately 50% increase. Although 1.0 w% CNC addition lowered the elastic modulus of the composite around 4% with respect to 0.5 w% CNC, maximum elastic modulus average, 1627.9 MPa (StDev = ± 107.3 MPa), was observed with the addition of 2.5 w% CNC, which is 79% increase with respect the neat PA6. The addition of 5.0 w% CNC and higher concentrations resulted in drop in the elastic modulus compared to 2.5 w% CNC composite. However, it is important to note that a sharp decrease (42%) in elastic modulus was observed in 7.5 w% CNC composite with respect to 5.0 w% CNC. It was observed that addition of 0.5 w% CNC (w.r.t. 0 w% CNC), 2.5 w% CNC (w.r.t. 1.0 w% CNC), and 7.5 w% CNC (w.r.t. 5.0 w% CNC) had resulted in statistically significant difference on the average elastic modulus as it can be seen from the data presented in Table 1. There is no significant difference between the neat PA6 and PA6 with concentrations higher than 7.5 w% CNC, thus, it can be noted the addition of CNC higher than 5.0 w% does not increase the elastic modulus of neat PA6.

The average tensile strength of the neat PA6 was measured as 38.8 MPa (StDev = ± 1.4 MPa) and 0.5 w% CNC addition increased the tensile strength of the composite approximately 15%, which is the highest increase in tensile strength of the composite with respect to the neat PA6. Statistically, 0.5 w% and 1.0 w% showed the highest average and there is no statistical difference between them. After this increase, the average tensile strength values of the composites started to drop; however, a sharp decrease in the tensile strength was observed in 7.5 w% CNC (15% w.r.t. 5.0 w% CNC and 17% w.r.t. neat PA6). It is important to note that the average tensile strength of 7.5, 10.0, and 15.0 w% CNC-PA6 was found to be lower than the neat PA6.

The average strain-at-break values of the composites at each concentration was measured lower than neat PA6 except for 1.0 w% CNC. The average strain-at-break of 1.0 w% CNC-PA6 was found to be 0.214 (StDev = ± 0.010), 3% higher than the average strain-at-break of neat PA6, which is not statistically different than the neat PA6. The lowest average strain-at-break was observed in 15 w% CNC.

Increase in the elastic modulus without loss in tensile strength at 0.5 w% and 2.5 w% shows effective strengthening impact of CNC on PA6 and suggest an efficient stress transfer between PA6 and CNC. However, decrease in the elastic modulus and tensile strength at 7.5 and higher w% of CNC loadings suggests that 7.5 w% CNC set the limit, and the agglomerates became detrimental to mechanical properties. Our findings in terms of the trend of elastic modulus and tensile strength are consistent with the results in Yousefian’s and Peng studies.^56^,^57^ It is important to note that, although the trends are similar, we observed 84% increase in elastics modulus (1629 MPa) with respect to the neat PA6 while Yousefian’s observed the maximum increase as 24% (1312 MPa) and Peng observed 31% (1540 MPa) increase. The main difference between studies is the manufacturing methods which suggests that solvent mixing method can provide a better CNC dispersion and stress transfer than the melt mixing according to the mechanical test results.

Tensile strength and strain-at-break showed similar trends as expected because both properties heavily depend on the imperfections and stress concentration points. Further, it can be argued that continues slight decrease in these properties with respect to CNC loading is also the result of the decrease in the polymer chain movement due to the increased particle content.

When the thickness of the samples is considered, any notch, agglomerate, and void can decrease strength and strain-at-break suddenly. As the concentration of CNC becomes higher than 1.0 w%, strain-at-break and tensile strength start dropping. This can be interpreted as a sign of agglomeration since agglomerates can form stress-concentration points. White regions in TEM image (Figure 8(c)) support the idea of imperfections in the sample because voids are seen as a white color under dark field TEM images. As noted in the literature,^58^ we can argue that the CNC particles are well-dispersed at low % CNC loadings; however, they tend to agglomerate at higher loading levels based on the mechanical properties and TEM images of the composites. According to the production method utilized in this study, the addition of CNC more than 5 w% is not recommended for the purpose of reinforcing.

### Modeling

**Figure 11.**
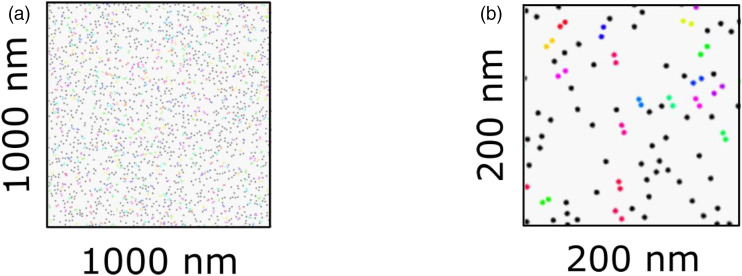
Locations of the fillers: general view (a) and zoomed-in view (b).

Figure 11(a) exhibits locations of 4.5 v % dispersed fillers in the defined space (*1μmx1μm*) as a result of the simulation. The distribution of fillers is based on the two main assumptions: locations of fillers are selected from the uniform distribution function, and fillers do not overlap. In the case of overlap, the code detects it and assigns a new location to the filler. In that way, the output is created without any overlapping fillers. The aim of dispersing fillers was to detect agglomerated fillers and establish the three phases to calculate the stiffness of the composites. In order to detect agglomerated fillers, the *critical distance* parameter *γ[D]* was utilized. Figure 11(b) shows a zoomed-in view of an output. While black points represent the locations of the free fillers (non-agglomerated fillers), the same colored fillers represent agglomerated fillers. As we can see from Figure 11, the *critical distance* directly determines the number of agglomerates and the filler concentration within each agglomerate. If we were to choose *γ[D] = 0* (surface contact), then all fillers would be free fillers (non-agglomerated), and the fillers cannot overlap in the program. In that case, the three-phase model would be converged to a two-phase model, and we would not be able to capture the mechanical response accurately. In that sense, choosing the right critical distance is crucial to predict the stiffness.

**Figure 12.**
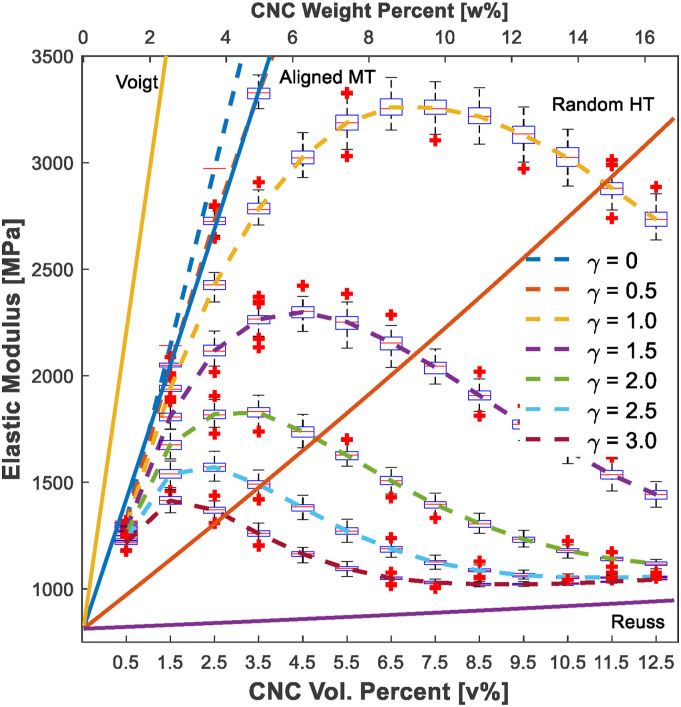
Elastic modulus versus CNC loading (v% and w% shown on the bottom and top *x*-axis, respectively) of the composite with different critical distances.

The predictions of the elastic modulus of the nanocomposites with different *critical distances* and CNC concentrations are given in Figure 12. One-hundred outcomes for each concentration are shown by box-plots where box represents 25–75% outcome and cross symbols represents outliers. As it can be seen in Figure 12, the modeling outputs can be as linear as the Mori–Tanaka prediction when we chose a *γ[D]* = 0, or they can evolve to the Reuss model around 10 v% loading when the *γ[D]* = 3. Two main factors play a key role here: *critical distance γ[D]* and the agglomeration property. The impact of the *critical distance* and agglomerates can be seen in Figure 12 when predictions of the proposed model the conventional models are compared. Sheng et al. studied FEA of polymer/clay composites and observed three states of the nanofillers as a result of their FEA stress analysis: “isolated,” “partly overlapped,” and “completely overlapped” particles.^59^ According to the scale of the FEA from their study, particles start to overlap between 10 and 20 nm, which corresponds *γ[D] =* 1–3 in this study. Load transfer efficiency is the lowest in completely overlapped particles. This can guide us to determine the *critical distance*. Musino et al.^41^ investigated closest distance (range is from 0.4 to 2.0 radius of the fillers) between fillers to define as an aggregate. None of the studies discussed how *critical distance* depends on the material. More investigation on what kinds of material parameters govern the *critical distance* should be conducted theoretically and experimentally.

Experiments in the literature exemplified a drop in the elastic modulus after around 4 w%,^60–63^ which is similar to the modeling results when *γ[D]* is approximately 2. In addition, the agglomeration property becomes dominant as its volume fraction increases. Since we used the Reuss model to calculate the stiffness of agglomerates, the results evolve to the Reuss model, and the trends show a drastic change as soon as it reaches around 5 w%, which actually represents the literature data.^64–68^ In these studies, the elastic modulus of the composites experimentally converged to a stable value (a plateau region) as the filler concentration increases or drops after a certain point of filler concentration. The decrease in the mechanical properties was related to agglomerates based on the analysis of SEM or TEM images. Thus, the proposed model has the capability of capturing the effect of agglomerates.

### Comparison of experiment and models

**Figure 13.**
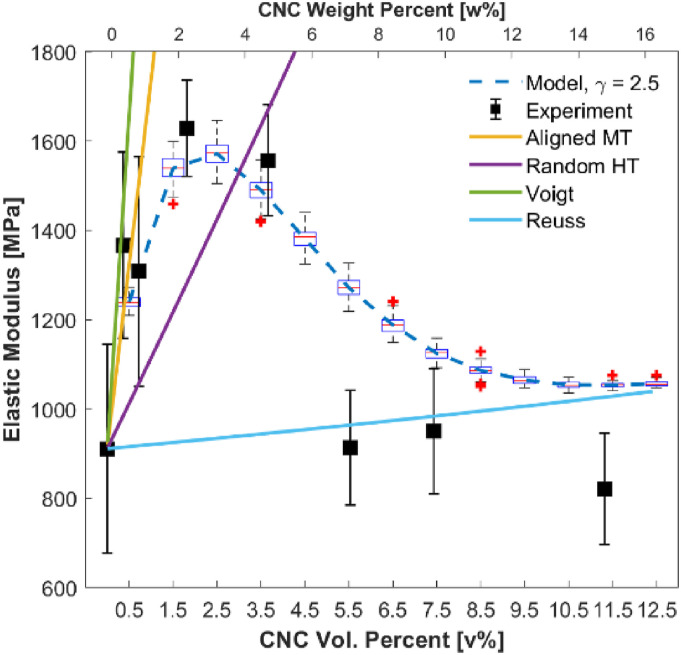
Elastic modulus versus CNC loading (v% and w% shown on the bottom and top *x*-axis, respectively) of models’ predictions and experimental results.

The longitudinal elastic modulus (*E*_*11*_) versus % CNC reinforced PA6 are shown in Figure 13. The figure includes proposed model predictions for *critical distance γ[D]* = 2.5, experimental results, and the results of conventional models (Mori–Tanaka, Halpin–Tsai, Voigt, and Reuss). The experimental results show a decrease in the elastic modulus of the samples after 5.00 w% CNC in PA6—the general trend is similar to studies in Refs. ^59, 67, and 68^. As discussed under the *Uniaxial Tensile Tests* section, some of the potential reasons for this drop can be due to agglomeration formation. The free filler may interact with the matrix more efficiently than agglomerates. The matrix may transfer the load non-uniformly to the agglomerates, the stress field of agglomerated fillers may overlap, and aspect ratio of the constitutes decreases. Each reason suggests the necessity of a model that contains agglomeration as a factor.

The trend of conventional models is unable to capture the experimental behavior due to the lack of an agglomeration parameter in their model development. On the other hand, the proposed model reveals a relatively good agreement with experimental results compared to conventional models. The distribution of fillers and theoretical discussion on critical distance should be studied in deep for a better understanding of nanocomposites.

A sensitivity analysis of the proposed model would provide a better understanding of the nanocomposite system and exhibit the capabilities of the model. Work is in progress to conduct a sensitivity study as the second stage of the current study.

## Conclusion

In this study, we evaluated the effect of nanofillers and agglomeration on the elastic modulus of a matrix by utilizing continuum-based analytical models. Nanocomposites were assumed to consist of three phases: the free filler, agglomeration, and matrix, which established the bases of the three-phase Mori–Tanaka model developed in this study. Among these phases, the agglomeration phase has experimentally and computationally been difficult to deal with due to the complexity and randomness of manufacturing and the nature of nanofillers. We incorporated a Monte Carlo method to disperse the fillers and detect the agglomerated fillers computationally to address previous modeling challenges. The critical distance was introduced as a parameter to identify and classify agglomerates and a hierarchical clustering method was employed. This provided a pathway to clearly define agglomerates and calculated volume fractions of the constitutes (matrix, free filler, agglomerated filler, and matrix). Based on the knowledge of agglomerated fillers and matrix and the necessary simplifications, the Reuss model was employed to calculate the stiffness of the agglomerates. The three-phase Mori–Tanaka model was then used to predict the elastic modulus of the nanocomposite. The prediction process was repeated one hundred times for each concentration to obtain reliable outcomes.

CNC reinforced PA6 samples were used as a model system and were manufactured with a spin-coating method. Detailed TEM characterization was performed in order to verify the proposed model. To our knowledge, this is the first TEM study that clearly displays CNC in a PA6 matrix. The predictions from the proposed model demonstrate a good agreement with the experimental results as opposed to the predictions from the conventional prediction models. As a summary, the current study contributes to the literature by (a) defining agglomerates computationally and showing their impact on the stiffness theoretically, (b) utilizing a statistical approach and continuum-based analytical models to capture the agglomeration effect on stiffness, and (c) verifying the proposed model and exploring the potentials of CNC as a reinforcement candidate. The proposed model can be implemented to various nanocomposites with necessary knowledge and/or assumptions for parameters such as orientation and distribution function of fillers. TEM can be utilized to obtain these parameters or machine learning can be applied to predict them. In conclusion, the importance and drawbacks of agglomerates on the stiffness of nanocomposites were revealed by this study.
